# Supplementary material for: Associations of Tumor Necrosis Factor-Alpha Gene Polymorphisms (TNF)-α TNF-863A/C (rs1800630), TNF-308A/G (rs1800629), TNF-238A/G (rs361525), and TNF-Alpha Serum Concentration with Age-Related Macular Degeneration
Source: Life (Basel). 2022 Jun 21;12(7):928. doi: 10.3390/life12070928 (PMC9319996; doi:10.3390/life12070928)
Supplement: Supplementary file 1 [file life-12-00928-s001.zip › life-1771036-supplementary.pdf]

Supplementary Materials

Table S1. Genotypes distribution of *TNF-α* SNPs in the control group using the Hardy-Weinberg equilibrium.

SNP	Group	Allele frequency		Distribution of Genotypes	HWE *p*-Value
**TNF-863A/C (rs1800630)**	Control	A (0.151)	C (0.849)	10/87/258	645
**TNF-308A/G (rs1800629)**	Control	A (0.115)	G (0.885)	4/74/277	146
**TNF-238A/G (rs361525)**	Control	A (0.056)	G (0.944)	1/38/316	16

SNP: single nucleotide polymorphism; HWE: Hardy-Weinberg equilibrium; *p*-value: significance level.

Table S2. Distributions of rs1800630, rs1800629, rs361525 genotypes and alleles in patients with early AMD and control groups.

SNP	Genotypes/ Alleles	Group		*p*-Value
		Control n (%) (n = 355)	Early AMD n (%) (n = 330)	
**TNF-863A/C (rs1800630)**	CC	258 (72.7)	235 (71.2)	0.594
	AC	87 (24.5)	81 (24.5)	
	AA	10 (2.8)	14 (4.2)	
	C	603 (84.9)	551 (83.5)	0.463
	A	107 (15.1)	109 (16.5)	
**TNF-308A/G (rs1800629)**	GG	277 (78.0)	267 (80.9)	0.275
	AG	74 (20.8)	56 (17.0)	
	AA	4 (1.1)	7 (2.1)	
	G	628 (88.5)	590 (89.4)	0.579
	A	82 (11.5)	70 (10.6)	
**TNF-238A/G (rs361525)**	GG	316 (89.0)	303 (91.8)	0.329
	AG	38 (10.7)	27 (8.2)	
	AA	1 (0.3)	0 (0.0)	
	G	670 (94.4)	633 (95.9)	0.186
	A	40 (5.6)	27 (4.1)	

SNP: single nucleotide polymorphism; AMD: age-related macular degeneration; *p*-value: significance level (statistically significant when $p < 0.05$).

Table S3. Distributions of rs1800630, rs1800629, rs361525 genotypes and alleles in patients with early and exudative AMD.

SNP	Genotypes/ Alleles	Group		*p*-Value
		Early AMD n (%) (n = 330)	Exudative AMD n (%) (n = 393)	
**TNF-863A/C (rs1800630)**	CC<br>AC<br>AA	235 (71.2)<br>81 (24.5)<br>14 (4.2)	263 (66.9)<br>108 (27.5)<br>22 (5.6)	0.420
	C<br>A	551 (83.5)<br>109 (16.5)	634 (80.7)<br>152 (19.3)	0.164
**TNF-308A/G (rs1800629)**	GG<br>AG<br>AA	267 (80.9)<br>56 (17.0)<br>7 (2.1)	297 (75.6)<br>91 (23.2)<br>5 (1.3)	0.090
	G<br>A	590 (89.4)<br>70 (10.6)	685 (87.2)<br>101 (12.8)	0.188
**TNF-238A/G (rs361525)**	GG<br>AG<br>AA	303 (91.8)<br>27 (8.2)<br>0 (0.0)	361 (91.9)<br>30 (7.6)<br>2 (0.5)	0.417
	G<br>A	633 (95.9)<br>27 (4.1)	752 (95.7)<br>34 (4.3)	0.825

SNP: single nucleotide polymorphism; AMD: age-related macular degeneration; *p*-value: significance level (statistically significant when $p < 0.05$).

Table S4. Binary logistic regression analysis of rs1800630, rs1800629, rs361525 in patients with early AMD and control groups.

Model	Genotype/Allele	OR (95% CI)	*p*-Value	AIC
**TNF-863A/C (rs1800630)**				
Additive	**A**	1.106 (0.836–1.464)	0.480	950.200
Codominant	**A/C**<br>**A/A**	1.022 (0.720–1.451)<br>1.537 (0.670–3.527)	0.902<br>0.310	951.654
Dominant	**A/C+A/A**	1.075 (0.770–1.501)	0.670	950.517
Recessive	**A/A**	1.528 (0.669–3.490)	0.314	949.669
Overdominant	**A/C**	1.002 (0.707–1.420)	0.991	950.699
**TNF-308A/G (rs1800629)**				
Additive	**A**	0.912 (0.654–1.271)	0.586	950.401

Codominant	**A/G**<br>**A/A**	0.785 (0.534–1.155)<br>1.816 (0.525–6.273)	0.219<br>0.346	950.099
Dominant	**A/G+A/A**	0.838 (0.578–1.216)	0.352	949.829
Recessive	**A/A**	1.902 (0.552–6.557)	0.309	949.619
Overdominant	**A/G**	0.776 (0.528–1.141)	0.197	949.023
**TNF-238A/G (rs361525)**				
Additive	**A**	0.709 (0.428–1.176)	0.183	948.898
Codominant	**A/G**<br>**A/A**	0.741 (0.441–1.244)<br>–	0.257<br>–	950.082
Dominant	**A/G+A/A**	0.722 (0.431–1.209)	0.215	949.145
Recessive	**A/A**	–	–	949.383
Overdominant	**A/G**	0.743 (0.443–1.248)	0.262	949.425

OR: odds ratio; CI: confidence interval; AIC: Akaike information criterion; *p*-value: significance level (statistically significant when $p < 0.05$).

Table S5. Binary logistic regression analysis of rs1800630, rs1800629, rs361525 in patients with exudative AMD and control groups.

**Model**	**Genotype/Allele**	**OR (95% CI)**	***p*-Value**	**AIC**
**TNF-863A/C (rs1800630)**				
Additive	**A**	1.268 (0.968–1.660)	0.085	984.183
Codominant	**A/C**<br>**A/A**	1.169 (0.831–1.645)<br>1.995 (0.907–4.389)	0.370<br>0.086	985.604
Dominant	**A/C+A/A**	1.255 (0.908–1.737)	0.169	985.293
Recessive	**A/A**	1.912 (0.873–4.184)	0.105	984.410
Overdominant	**A/C**	1.125 (0.802–1.579)	0.494	986.720
**TNF-308A/G (rs1800629)**				
Additive	**A**	1.217 (0.878–1.687)	0.239	985.791
Codominant	**A/G**<br>**A/A**	1.235 (0.861–1.772)<br>1.317 (0.338–5.130)	0.251<br>0.692	987.754
Dominant	**A/G+A/A**	1.240 (0.871–1.765)	0.233	985.762
Recessive	**A/A**	1.254 (0.323–4.872)	0.743	989.081
Overdominant	**A/G**	1.230 (0.858–1.763)	0.260	985.912
**TNF-238A/G (rs361525)**				

Additive	**A**	0.734 (0.454–1.184)	0.205	985.570
Codominant	**A/G**<br>**A/A**	0.649 (0.386–1.091)<br>2.043 (0.171–24.360)	0.103<br>0.572	986.134
Dominant	**A/G+A/A**	0.681 (0.410–1.133)	0.139	984.985
Recessive	**A/A**	2.121 (0.178–25.257)	0.552	986.813
Overdominant	**A/G**	0.647 (0.385–1.088)	0.101	984.471

OR: odds ratio; CI: confidence interval; AIC: Akaike information criterion; *p*-value: significance level (statistically significant when $p < 0.05$).

Table S6. Haplotype association with the predisposition to early AMD occurrence.

**TNF -863A/C (rs1800630)**	**TNF -308A/G (rs1800629)**	**TNF -238A/G (rs361525)**	**Frequency**		**OR (95 % CI)**	***p*-Value**
			**Control**	**Early AMD**		
C	G	G	0.691	0.706	1.00	–
A	A	G	0.134	0.137	0.96 (0.66–1.40)	0.83
C	A	G	0.104	0.09	0.84 (0.58–1.21)	0.35
C	G	A	0.056	0.036	0.70 (0.42–1.18)	0.18
A	G	G	0.016	0.025	1.58 (0.66–3.78)	0.31
A	A	A	0	0.005	–	–

OR: odds ratio; CI: confidence interval; *p*-value: significance level (statistically significant when $p < 0.05$).

Table S7. Distributions of rs1800630, rs1800629, rs361525 genotypes and alleles in patients with early AMD and control groups by gender.

**SNP**	**Geno-types/ Alleles**	**Female**		***p*-Value**	**Male**		***p*-Value**
		**Control n (%) (n = 222)**	**Early AMD n (%) (n = 227)**		**Control n (%) (n = 133)**	**Early AMD n (%) (n = 103)**	
**TNF-863A/C (rs1800630)**	CC<br>AC<br>AA	163 (73.4)<br>50 (22.5)<br>9 (4.1)	169 (74.4)<br>49 (21.6)<br>9 (4.0)	0.969	95 (71.4)<br>37 (27.8)<br>1 (0.8)	66 (64.1)<br>32 (31.1)<br>5 (4.9)	0.105
	C<br>A	376 (84.7)<br>68 (15.3)	387 (85.2)<br>67 (14.8)	0.815	227 (85.3)<br>39 (14.7)	164 (79.6)<br>42 (20.4)	0.102

**TNF-308A/G (rs1800629)**	GG<br>AG<br>AA	174 (78.4)<br>46 (20.7)<br>2 (0.9)	185 (81.5)<br>37 (16.3)<br>5 (2.2)	0.280	103 (77.4)<br>28 (21.1)<br>2 (1.5)	82 (79.6)<br>19 (18.4)<br>2 (1.9)	0.861
	G<br>A	394 (88.7)<br>50 (11.3)	407 (89.6)<br>47 (10.4)	0.661	234 (88.0)<br>32 (12.0)	183 (88.8) 23<br>(11.2)	0.771
**TNF-238A/G (rs361525)**	GG<br>AG<br>AA	199 (89.6)<br>22 (9.9)<br>1 (0.5)	207 (91.2)<br>20 (8.8)<br>0 (0.0)	0.550	117 (88.0)<br>16 (12.0)<br>0 (0.0)	96 (93.2)<br>7 (6.8)<br>0 (0.0)	0.179
	G<br>A	420 (94.6)<br>24 (5.4)	434 (95.6)<br>20 (4.4)	0.488	250 (94.0)<br>16 (6.0)	199 (96.6)<br>7 (3.4)	0.190

SNP: single nucleotide polymorphism; AMD: age-related macular degeneration; *p*-value: significance level (statistically significant when $p < 0.05$).

Table S8. Binary logistic regression analysis of rs1800630, rs1800629, rs361525 in the early AMD and control groups by gender.

**Model**	**Genotype/Allele**	**OR (95 proc. CI)**	***p*-Value**	**AIC**
**Female**				
**TNF-863A/C (rs1800630)**				
Additive	**A**	0.962 (0.683–1.356)	0.826	624.342
Codominant	**A/C**<br>**A/A**	0.945 (0.603–1.481)<br>0.964 (0.374–2.491)	0.806<br>0.940	626.328
Dominant	**A/C+A/A**	0.948 (0.622–1.445)	0.804	624.329
Recessive	**A/A**	0.977 (0.381–2.509)	0.962	624.388
Overdominant	**A/C**	0.947 (0.606–1.480)	0.811	624.333
**TNF-308A/G (rs1800629)**				
Additive	**A**	0.913 (0.604–1.381)	0.667	624.206
Codominant	**A/G**<br>**A/A**	0.757 (0.468–1.222)<br>2.351 (0.450–12.278)	0.254<br>0.311	623.803
Dominant	**A/G+A/A**	0.823 (0.518–1.308)	0.409	623.709
Recessive	**A/A**	2.477 (0.476–12.905)	0.281	623.109
Overdominant	**A/G**	0.745 (0.462–1.203)	0.229	622.933
**TNF-238A/G (rs361525)**				
Additive	**A**	0.806 (0.438–1.483)	0.488	623.906

Codominant	**A/G**<br>**A/A**	0.874 (0.463–1.651)<br>–	0.678<br>–	624.807
Dominant	**A/G+A/A**	0.836 (0.445–1.570)	0.577	624.079
Recessive	**A/A**	–	–	–
Overdominant	**A/G**	0.878 (0.465–1.659)	0.689	624.230
**Male**				
**TNF-863A/C (rs1800630)**				
Additive	**A**	1.512 (0.925–2.470)	0.099	322.597
Codominant	**A/C**<br>**A/A**	1.245 (0.705–2.197)<br>7.197 (0.822–63.026)	0.450<br>0.075	322.640
Dominant	**A/C+A/A**	1.402 (0.808–2.432)	0.230	323.900
Recessive	**A/A**	6.735 (0.774–58.566)	0.084	321.210
Overdominant	**A/C**	1.169 (0.665–2.055)	0.587	325.046
**TNF-308A/G (rs1800629)**				
Additive	**A**	0.921 (0.526–1.614)	0.775	325.260
Codominant	**A/G**<br>**A/A**	0.852 (0.445–1.634)<br>1.256 (0.173–9.110)	0.630<br>0.822	327.042
Dominant	**A/G+A/A**	0.879 (0.469–1.649)	0.688	325.180
Recessive	**A/A**	1.297 (0.180–9.367)	0.797	325.275
Overdominant	**A/G**	0.848 (0.443–1.624)	0.619	325.093
**TNF-238A/G (rs361525)**				
Additive	**A**	0.533 (0.211–1.349)	0.184	323.474
Codominant	**A/G**<br>**A/A**	0.533 (0.211–1.349)<br>–	0.184<br>–	325.474
Dominant	**A/G+A/A**	0.533 (0.211–1.349)	0.184	323.474
Recessive	**A/A**	–	–	–
Overdominant	**A/G**	0.533 (0.211–1.349)	0.184	323.474

OR: odds ratio; CI: confidence interval; AIC: Akaike information criteria; *p*-value: significance level (statistically significant when $p < 0.05$).

Table S9. Binary logistic regression analysis of rs1800630, rs1800629, rs361525 in exudative AMD and control groups by gender.

Model	Genotype/Allele	OR (95 proc. CI)	*p*-Value	AIC

**Female**				
**TNF-863A/C (rs1800630)**				
Additive	**A**	1.288 (0.919–1.803)	0.141	607.226
Codominant	**A/C**<br>**A/A**	1.411 (0.908–2.191)<br>1.364 (0.558–3.334)	0.126<br>0.496	608.827
Dominant	**A/C+A/A**	1.403 (0.927–2.123)	0.109	604.832
Recessive	**A/A**	1.240 (0.511–3.008)	0.634	609.190
Overdominant	**A/C**	1.381 (0.893–2.138)	0.147	607.299
**TNF-308A/G (rs1800629)**				
Additive	**A**	1.304 (0.847–2.007)	0.228	607.948
Codominant	**A/G**<br>**A/A**	1.390 (0.875–2.207)<br>0.818 (0.102–6.531)	0.163<br>0.850	609.387
Dominant	**A/G+A/A**	1.363 (0.864–2.148)	0.183	607.629
Recessive	**A/A**	0.762 (0.096–6.044)	0.797	609.353
Overdominant	**A/G**	1.393 (0.878–2.210)	0.160	607.423
**TNF-238A/G (rs361525)**				
Additive	**A**	0.761 (0.401–1.442)	0.402	608.712
Codominant	**A/G**<br>**A/A**	0.797 (0.411–1.542)<br>–	0.500<br>–	610.218
Dominant	**A/G+A/A**	0.775 (0.403–1.492)	0.446	608.837
Recessive	**A/A**	–	–	608.673
Overdominant	**A/G**	0.799 (0.413–1.547)	0.506	608.976
**Male**				
**TNF-863A/C (rs1800630)**				
Additive	**A**	1.173 (0.741–1.857)	0.495	375.241
Codominant	**A/C**<br>**A/A**	0.835 (0.480–1.452)<br>7.129 (0.854–59.491)	0.523<br>0.070	372.042
Dominant	**A/C+A/A**	0.995 (0.585–1.692)	0.985	375.709
Recessive	**A/A**	7.481 (0.901–62.117)	0.062	370.452
Overdominant	**A/C**	0.786 (0.453–1.362)	0.390	374.970
**TNF-308A/G (rs1800629)**				
Additive	**A**	1.108 (0.668–1.837)	0.691	375.551

Codominant	**A/G**<br>**A/A**	1.042 (0.580–1.870)<br>1.679 (0.272–10.382)	0.891<br>0.577	377.382
Dominant	**A/G+A/A**	1.081 (0.613–1.906)	0.787	375.636
Recessive	**A/A**	1.664 (0.270–10.243)	0.583	375.401
Overdominant	**A/G**	1.029 (0.574–1.844)	0.923	375.700
**TNF-238A/G (rs361525)**				
Additive	**A**	0.739 (0.356–1.532)	0.416	375.042
Codominant	**A/G**<br>**A/A**	0.482 (0.203–1.144)<br>–	0.098<br>–	372.360
Dominant	**A/G+A/A**	0.588 (0.260–1.330)	0.202	374.053
Recessive	**A/A**	–	–	–
Overdominant	**A/G**	0.474 (0.200–1.125)	0.090	372.719

OR: odds ratio; CI: confidence interval; AIC: Akaike information criteria; p-value: significance level (statistically significant when $p < 0.05$).

Table S10. Serum TNF-α levels in relation to the genotypes of *TNF-α* polymorphisms.

**Model**		**All Subjects (pg/mL), Median (IQR)**	***p*-Value**	**Exudative AMD (pg/mL), median (IQR)**	***p*-Value**	**Control Group (pg/mL), Median (IQR)**	***p*-Value**
**TNF-863A/C (rs1800630)**							
Dominant	AC+AA<br>vs.<br>CC	23.51 (12.25)<br>vs.<br>23.91 (8.49)	0.908	15.43 (7.45)<br>vs.<br>16.56 (5.63)	0.724	33.21 (9.52)<br>vs.<br>29.8 (5.03)	0.309
**TNF-308A/G (rs1800629)**							
Dominant	AG+AA<br>vs.<br>GG	21.69 (8.35)<br>vs.<br>24.19 (9.83)	0.563	16.69 (3.88)<br>vs.<br>16.04 (6.71)	0.858	31.7 (1.64)<br>vs.<br>30.54 (6.66)	0.812
**TNF-238A/G (rs361525)**							
Dominant	AG+AA<br>vs.<br>GG	18.6 (5.6)<br>vs.<br>24.24 (9.74)	0.333	17.12 (7.04)<br>vs.<br>16.07 (6.22)	0.825	– (–)<br>vs.<br>31.13 (6.11)	–

p-value: significance level (statistically significant when $p < 0.05$).
